# Supplementary material for: Sustainability Assessment of Combined Animal Fodder and Fuel Production from Microalgal Biomass
Source: Int J Environ Res Public Health. 2021 Oct 28;18(21):11351. doi: 10.3390/ijerph182111351 (PMC8583398; doi:10.3390/ijerph182111351)
Supplement: Supplementary file 1 [file ijerph-18-11351-s001.zip › SI1.pdf]

Supporting Information

1 Modifications to the AF+energy model

A detailed description of the AF+energy system is given in [2] and not repeated here. The corresponding process model is available free of charge from the supporting information therein. For this study, the model was modified as follows:

1. The input *market for land use change, annual crop, ES* [3] was moved from the cultivation subprocess to the infrastructure subprocess. Additional inputs were added to model the transformation of land from natural to industrial area, and the occupation of land by an industrial area. This was necessary to evaluate the impact of algae cultivation on the PEF 2.0 indicator *resources, land use* [1].

2. The evaporation of water from the cultivation plant was previously modeled by a biosphere flow *water to air, low population density, long-term* [2]. As all water in the cultivation stage is ocean water, evaporation should not contribute a water dissipation impact according to the the PEF 2.0 guidelines [1]. Hence, this flow was removed. For the same reason, the wastewater treatment process *treatment of wastewater, average, capacity 1.1E10l/year, CH* [3] was adapted to eliminate evaporation. The spray-drying process of the AF+fodder system retains a minor evaporation impact equivalent to the amount of freshwater added (addition of tap water in accordance with Ecoinvent activity *market for tap water, Europe without Switzerland* [3]).

3. The combustion of locally produced biogas in the AF+energy system was shifted from the residue valorization (RV) model to the biogas CHP. Thus instead of heat and electricity, the new RV model produces biogas, which substitutes market biogas. The biospheric oxygen input necessary for combustion was also removed. This was done to model biogas combustion consistently and to improve the clarity of the RV model.

4. In the former AF+energy model, nutrients recycled from anaerobic digestate were modeled implicitly by reducing the cultivation nutrient demand [2]. In the updated model, this is explicitly modeled in the form of outputs of urea, TSP and captured CO_2 (where the latter is equivalent to reduced biogas CHP operation). Note that the net nutrient demand per unit biomass did not change. This change was merely done to improve the clarity of the RV model.

5. The treatment of digestate in the former AF+energy model, previously modeled by Ecoinvent activity *treatment of wastewater, average, capacity 1.1E10l/year*, was omitted in the new version as it did not influence the results [2]. Note that the RV model's input requirements for electricity and heat remain unchanged, as did the outflow of methane to the atmosphere (leakage).

6. Building infrastructure was added to the anaerobic digestion model via the input *market for chemical factory, organics, GLO* [3] in equal amount

to the spray-drying model. This was done to improve the comparability of both RV models, particularly in PEF 2.0 category *resources, minerals and metals* [1].

The effect of the update on the environmental LCIA scores is presented in Table 1.

Table 1: Effect of modifications on LCIA results

impact category	original model [2]	modified model (this study)	rel. difference
Climate change biogenic	2.65E-02	2.52E-02	-4.9%
Climate change fossil	6.50E-02	6.41E-02	-1.5%
Climate change land use and land use change	2.34E-03	2.77E-03	15.5%
climate change total	9.38E-02	9.21E-02	-1.9%
Freshwater and terrestrial acidification	1.42E-03	1.13E-03	-26.6%
Freshwater ecotoxicity	5.57E-01	5.91E-01	5.7%
Freshwater eutrophication	2.46E-04	3.15E-04	22.0%
Marine eutrophication	2.05E-03	2.07E-03	0.9%
Terrestrial eutrophication	4.58E-03	3.11E-03	-47.1%
Carcinogenic effects	1.36E-08	2.02E-08	32.5%
Ionising radiation	-1.33E-02	-9.73E-03	-36.2%
Non-carcinogenic effects	1.82E-07	1.85E-07	1.8%
Ozone layer depletion	2.19E-08	5.12E-08	57.2%
Photochemical ozone creation	4.30E-04	4.84E-04	11.0%
Respiratory effects, inorganics	1.31E-08	9.49E-09	-37.6%
Dissipated water	2.53E+00	2.56E-01	-888.1%
Fossils	2.05E-01	2.95E-01	30.7%
Land use	1.61E+02	6.73E+01	-138.8%
Minerals and metals	5.63E-06	2.27E-06	-147.9%

References

[1] S. Fazio, V. Castellani, S. Sala, E. M. Schau, M. Secchi, L. Zampori, and E. Diaconu. Supporting information to the characterisation factors of recommended ef life cycle impact assessment methods, 2018.

[2] Benjamin W. Portner, Christian H. Endres, Thomas Brück, and Daniel Garbe. Life cycle greenhouse gas emissions of microalgal fuel from thin-layer cascades. *Bioprocess and biosystems engineering*, 25:294, 2021.

[3] Gregor Wernet, Christian Bauer, Bernhard Steubing, Jürgen Reinhard, Emilia Moreno-Ruiz, and Bo Weidema. The ecoinvent database version

3 (part i): overview and methodology. *The International Journal of Life Cycle Assessment*, 21(9):1218–1230, 2016.
